# AlphaPeptStats: an open-source Python package for automated and scalable statistical analysis of mass spectrometry-based proteomics

**DOI:** 10.1093/bioinformatics/btad461

**Published:** 2023-08-01

**Authors:** Elena Krismer, Isabell Bludau, Maximilian T Strauss, Matthias Mann

**Affiliations:** Department of Clinical Proteomics, Novo Nordisk Foundation Center for Protein Research, Faculty of Health Sciences, University of Copenhagen, 2200 Copenhagen, Denmark; Department of Proteomics and Signal Transduction, Max Planck Institute of Biochemistry, 82152 Martinsried, Germany; Department of Clinical Proteomics, Novo Nordisk Foundation Center for Protein Research, Faculty of Health Sciences, University of Copenhagen, 2200 Copenhagen, Denmark; Department of Clinical Proteomics, Novo Nordisk Foundation Center for Protein Research, Faculty of Health Sciences, University of Copenhagen, 2200 Copenhagen, Denmark; Department of Proteomics and Signal Transduction, Max Planck Institute of Biochemistry, 82152 Martinsried, Germany

## Abstract

**Summary:**

The widespread application of mass spectrometry (MS)-based proteomics in biomedical research increasingly requires robust, transparent, and streamlined solutions to extract statistically reliable insights. We have designed and implemented AlphaPeptStats, an inclusive Python package with currently with broad functionalities for normalization, imputation, visualization, and statistical analysis of label-free proteomics data. It modularly builds on the established stack of Python scientific libraries and is accompanied by a rigorous testing framework with 98% test coverage. It imports the output of a range of popular search engines. Data can be filtered and normalized according to user specifications. At its heart, AlphaPeptStats provides a wide range of robust statistical algorithms such as *t*-tests, analysis of variance, principal component analysis, hierarchical clustering, and multiple covariate analysis—all in an automatable manner. Data visualization capabilities include heat maps, volcano plots, and scatter plots in publication-ready format. AlphaPeptStats advances proteomic research through its robust tools that enable researchers to manually or automatically explore complex datasets to identify interesting patterns and outliers.

**Availability and implementation:**

AlphaPeptStats is implemented in Python and part of the AlphaPept framework. It is released under a permissive Apache license. The source code and one-click installers are freely available and on GitHub at https://github.com/MannLabs/alphapeptstats.

## 1 Introduction

Mass spectrometry (MS)-based proteomics has emerged as a powerful tool in biomedical research (Aebersold and Mann 2016). The rapid development of platforms and algorithms allows the identification and quantification of proteins with ever greater depth and precision. These workflows and search engines produce tables of identified and quantified proteins, which then require rigorous statistical methods to identify robust patterns and potentially biologically interesting outliers.

To date, we and others have developed popular applications, such as MSstats (Choi *et al.* 2014), Perseus (Tyanova *et al.* 2016), and MSPypeline (Heming *et al.* 2022) for the downstream analysis of proteomics data. While these tools mostly cover the required steps in the analysis pipeline, they can be limited in the search engines they support, access to the source code, test coverage, automation, and the ability to easily implement the latest algorithms. Furthermore, some of their functionality can readily be leveraged by domain experts, but this is more challenging for non-experts who need to integrate biological knowledge and contextualize the findings. This constitutes the need for an easy-to-use, rigorous, and robust tool to maximize the biological insight that can be extracted from quantitative proteomics data.

## 2 The AlphaPeptStats library

As part of our recently developed AlphaPept framework (Strauss *et al.* 2021, Voytik *et al.* 2022, Zeng *et al.* 2022), we implemented AlphaPeptStats in Python because of its straightforward syntax and the availability of high-quality scientific libraries. AlphaPeptStats is built on top of highly performant, widely used, and community-tested computing packages such as NumPy (Harris *et al.* 2020), Plotly (https://plotly.com/), Pandas (McKinney 2010), and SciPy (Virtanen *et al.* 2020). We additionally implemented state-of-the-art bioinformatic libraries, such as diffxpy from the *Scanpy-package* for differential expression analysis (Wolf *et al.* 2018), *a GO tool* for enrichment analysis with gene ontology (GO)-terms, tailored for MS (Schölz *et al.* 2015), and pyteomics allowing among other things the import of various proteomics data formats (Goloborodko *et al.* 2013, Levitsky *et al.* 2018).

The AlphaPeptStats source code is freely available on GitHub under the permissive Apache license. The package can readily be installed from PyPI using the standard pip module. Additionally, we provide one-click installers for Linux, MacOS, and Windows and a Dockerfile for containerized deployment, e.g. in cloud environments. Furthermore, automated postprocessing workflows can be created in AlphaPeptStats. This can also be used to systematically iterate over available options such as different normalization methods to identify best-performing ones.

Additionally, we have deployed a web-based version of AlphaPeptStats that is hosted on Streamlit-share (see GitHub repository for the link) from Streamlit (https://streamlit.io/). This enables users to explore and use AlphaPeptStats without requiring the installation of any software.

Proteomics has a long history of open-source proteomic tools, such as Trans-Proteomic Pipeline (Deutsch *et al.* 2010), Skyline (MacLean *et al.* 2010), OpenMS (Röst *et al.* 2016), FlashLFQ (Millikin *et al.* 2018), and Proline (Bouyssié *et al.* 2020). We designed AlphaPeptStats with best software engineering practices in mind, including continuous integration pipelines on GitHub, ensuring that the software is continuously tested and verified. To monitor the test coverage of our library, we employed Codecov (https://about.codecov.io), which allows us to gauge the percentage of executed source code by the test suite.

Our extensive testing framework reports a test coverage of 98%, providing confidence in accuracy and reliability of the software, in line with standard packages such as NumpPy or Pandas.

Extensive documentation of the AlphaPeptStats functionalities was a key part of this project and include several Jupyter notebooks that serve as tutorials to guide novice users. These notebooks are designed to encourage user engagement and offer a step-by-step approach to learning the package, Alternatively, the graphical user interface is straightforward to learn as well as allowing quick and easy data exploration.

## 3 Overview of the AlphaPeptStats workflow

**Figure 1.**
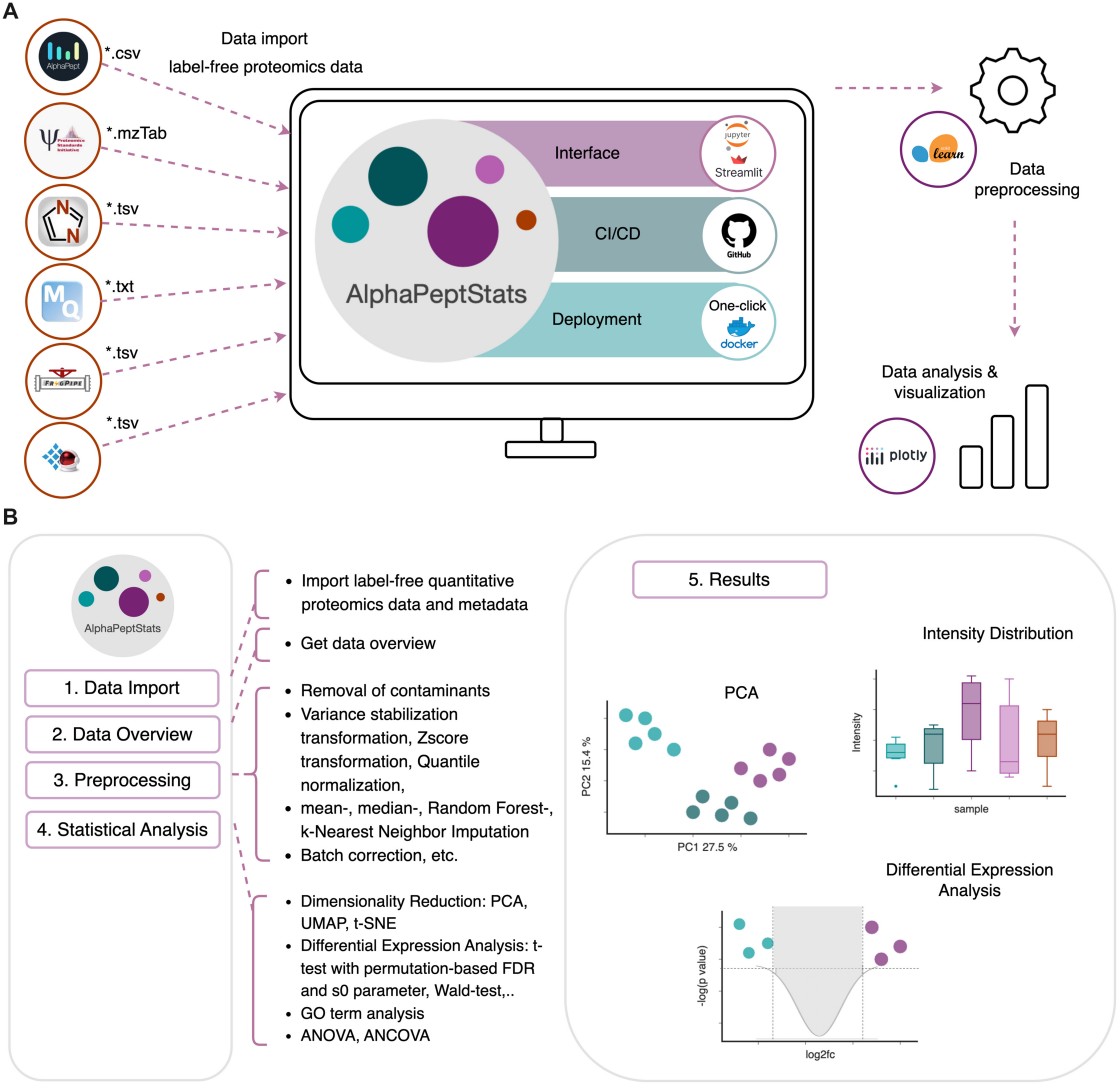
Overview of AlphaPeptStats and its ecosystem. (A) AlphaPeptStats relies on several community-tested packages. It supports the import of AlphaPept, DIA-NN, MaxQuant, Spectronaut, and FragPipe files and also enables import of data in the generic mzTab-format. It can be interfaced with a GUI via streamlit and or can be used as a Python library, e.g. by loading and using in Jupyter Notebooks. The source code is publicly available on GitHub with GitHub actions being used for continuous integration (CI) and continuous delivery (CD). The application with GUI can be easily installed via a one-click installer or deployed via Docker, ensuring flexibility and portability. For efficient data processing, analysis, and visualization, AlphaPeptStats utilizes various scientific computing packages, such as scikit-learn and plotly, in addition to other relevant tools. (B) Symbolic display of the graphical user interface of AlphaPeptStats, depicting its step-wise workflow and highlighting its comprehensive functionalities enabling meaningful interpretation of data.

At present, AlphaPeptStats is already capable of importing and processing label-free proteomics data generated from multiple software platforms, including MaxQuant (Cox and Mann 2008), AlphaPept, DIA-NN (Demichev *et al.* 2020), Spectronaut (Bruderer *et al.* 2015), and the FragPipe computational framework (Kong *et al.* 2017, Teo *et al.* 2021, Yu *et al.* 2020, 2021, da Veiga Leprevost *et al.* 2020). Additionally, it supports the mzTab data exchange format for proteomics experiments (Griss *et al.* 2014) (Fig. 1A). The modular design of the import functions allow straightforward extensions to other data formats.

Users are required to specify their proteomics results file and accompanying metadata. AlphaPeptStats provides a high-level API by storing data in a Python class named DataSet, with multiple methods ranging from data preprocessing, statistical analysis, GO analysis, and to visualization. The latter can export vector graphics for subsequent use in publications. An overview of the processing steps in AlphaPeptStats is provided in Fig. 1B as well as in Supplementary Table S1.

### 3.1 Preprocessing

After loading the data into a DataSet object, the user can select multiple optional preprocessing steps ranging from the removal of contaminants, normalization to imputation. For contaminant removal, AlphaPeptStats uses a recently developed library (Frankenfield *et al.* 2022) to help decrease false discoveries. In addition, AlphaPeptStats incorporates various normalization and imputation techniques to facilitate robust and accurate data analysis. One of the methods that we integrated—random forest imputation—has demonstrated superior performance compared to other commonly used imputation methods in several studies (Jin *et al.* 2021, Kokla *et al.* 2019). This algorithm was imported from Scikit-Learn, demonstrating how easily state-of-the-art methods can be added to AlphaPeptStats.

Importantly, all selected preprocessing steps are stored in the DataSet object, ensuring reproducibility. Different normalization and imputation methods can be systematically assessed as AlphaPeptStats can iterate through them automatically by means of passing a single parameter to the plotting functions.

### 3.2 Visualization and statistical analysis

Users can visualize their results via dedicated functions that allow the straightforward interpretation of the data, including principal component analysis plots, heatmaps, dendrogram, and volcano plots. Figures can be exported as publication-ready scalable vector graphics. AlphaPeptStats leverages the capabilities of the Plotly graphing library, producing interactive and zoomable graphs by default and enabling advanced users to tailor the generated figures to their specific needs and preferences.

Statistical testing for differential expression analysis can be performed using Analysis of Variance (ANOVA), Analysis of Covariance (ANCOVA), or t-testing. We further provide a reimplementation from R of the significance analysis of microarrays (SAM), which is a very widely used algorithm in proteomic (Tusher *et al.* 2001). Significantly expressed proteins can then be subjected to GO annotation.

### 3.3 Graphical user interface

As AlphaPeptStats is a Python library it can be imported and used in any Python program, scripts, or Jupyter Notebooks. As mentioned, figures are produced by the incorporated Plotly library, making graphs interactively explorable.

Furthermore, the popular Streamlit library provides even easier access to AlphaPeptStats functionalities and output for non-coders. In this case, the graphical user interface of AlphaPeptStats enables users to directly select functions and analyze their data in a browser-based environment.

## 4 Application of AlphaPeptStats

To illustrate the capabilities of AlphaPeptStats, we applied it to our recently published study of non-alcoholic liver disease (Niu *et al.* 2019). AlphaPeptStats facilitated a comprehensive downstream analysis, including preprocessing, data visualization, and extended biomarker discovery across different disease groups, all in a simple Jupyter notebook format. Additionally, we assessed the performance of our library using a standardized spiked proteomics dataset (Ramus *et al.* 2016). This standardized spiked proteomics dataset allowed us to evaluate the performance of AlphaPeptStats with simulated and ground truth data. Our analysis confirmed that random forest performed best, whereas mean imputation, for instance, led to a higher percentage of false positives (best Area Under the Curve (AUC) 1.0 versus 0.904). Analyses were performed with AlphaPeptStats version 0.6.2 and accompanying notebooks can be found as Supplementary Notebook S1 and Supplementary Notebook S2.

## 5 Conclusion

We developed AlphaPeptStats, a user-friendly, open-source package dedicated to the protein-centric downstream analysis of mass spectrometry data, covering all steps from preprocessing and statistical analysis to visualization. Apart from stand-alone use, it can also easily be incorporated into automated bioinformatics pipelines. It features extensive tests and robust design principles of software engineering on GitHub, such as continuous testing and continuous integration to ensure a stable and reliable workflow. Its modular framework allows extensions with additional functionality, such as the analysis of isotopically labeled data. We envision that AlphaPeptStats will be a suitable standard for statistical analysis and exploration for the challenging proteomics data set that can readily be produced today.

## Supplementary Material

btad461_Supplementary_Data
