# Supplementary material for: AlphaPeptStats: an open-source Python package for automated and scalable statistical analysis of mass spectrometry-based proteomics
Source: Bioinformatics. 2023 Aug 1;39(8):btad461. doi: 10.1093/bioinformatics/btad461 (PMC10415174; doi:10.1093/bioinformatics/btad461)
Supplement: btad461_Supplementary_Data [file btad461_supplementary_data.zip › supplementary_manual.pdf]

alphastats

Release 0.6.2

Elena Krismer

Jun 18, 2023

CONTENTS:

1 Installation 3
1.1 Pip . . . 3

2 Data import 5
2.1 Importing data from a Proteomics software . . . 5
2.2 Preparing metadata . . . 7
2.3 Creating a DataSet . . . 7

3 Data preprocessing 9
3.1 Removal of contaminations . . . 9
3.2 Normalization . . . 9
3.3 Imputation . . . 10
3.4 Subset Data . . . 10
3.5 Remove Samples . . . 10

4 Functions 11
4.1 Preprocessing . . . 11
4.2 Figures . . . 11
4.3 Statistical Analysis . . . 12
4.4 GO Analysis . . . 12
4.5 Misc . . . 12

5 GO Analysis 13
5.1 Requirements . . . 13

6 API reference for alphastats 15
6.1 File Loader . . . 15
6.2 DataSet . . . 16

7 Indices and tables 25

Python Module Index 27

Index 29

AlphaPeptStats is an open-source package for analyzing mass spectrometry-based proteomics data.

AlphaPeptStats was developed to in order to simplify and standardize the process of analyzig complex datasets. Hereby AlphaPeptStats supports proteomics data generated by *AlphaPept*, *DIA-NN*, *FragPipe* and *MaxQuant*. The tool allows a structured workflow like importing data, preprocessing data and visualization.

AlphaPeptStats is an open-source package for analyzing mass spectrometry-based proteomics data.

AlphaPeptStats was developed to simplify and standardize the process of analyzing complex datasets. Hereby AlphaPeptStats supports proteomics data generated by `AlphaPept`, `DIA-NN`, `FragPipe`, `MaxQuant` and `Spectronaut` and quantiative proteomics results in `mzTab` format. The tool allows a structured workflow from importing data, preprocessing data to visualization.

AlphaPeptStats was developed by the Mann Group at the University of Copenhagen and is freely available with an Apache License. External Python packages (available in the requirements folder) have their own licenses, which can be consulted on their respective websites.

The workflow consists of:

1. Import of proteomics data
2. Creation of the DataSet, consisting of the imported proteomics data and metadata
3. Data preprocessing (optional)
4. Statistical Analysis and Visualization

CHAPTER

ONE

INSTALLATION

1.1 Pip

For any Python package, it is highly recommended to use a separate conda virtual environment, as otherwise, dependency conflicts can occur with already existing packages.

```
conda create --name alphastats python=3.8
conda activate alphastats
```

AlphaStats can be installed in an existing Python 3.8 environment with a single `bash` command.

```
pip install alphastats
```

CHAPTER TWO

DATA IMPORT

Currently, AlphaStats allows the analysis of five quantitative proteomics software packages: AlphaPept, DIA-NN, FragPipe, MaxQuant and Spectronaut. As the output of these software differs significantly data needs to be loaded in customized loaders.

Imported proteomics data and metadata can be combined in a DataSet, which will be used for the downstream analysis.

```
import alphastats

maxquant_data = alphastats.MaxQuantLoader(
    file="testfiles/maxquant_proteinGroups.txt"
)

dataset = alphastats.DataSet(
    loader = maxquant_data,
    metadata_path="../testfiles/maxquant/metadata.xlsx",
    sample_column="sample"
)
```

2.1 Importing data from a Proteomics software

To import data from these software the columns describing intensity and Protein/ProteinGroups have to be specified when loading. Each loader has a default for the `intensity_column`and the `index_column`, however, the column naming can vary depending on the version of the software and personalized settings.

As we are dealing with wide data, a column represents the intensity for one sample. Thus the `intensity_column` must be specified as follow: For MaxQuant `"LFQ intensity [sample]"` or for AlphaPept `"[sample]_LFQ"` (this is already set as default).

Upon data import, the proteomics data gets processed in an internal format.

2.1.1 Additional modifications by AlphaStats

When importing the data, AlphaStats will identify potential contaminations based on a contaminant library, created by Frankenfield et al. 2022. This information will be added as an extra column to the imported data and can either be ignored or used for filtering in the preprocessing step.

2.1.2 AlphaPept

Alphapept output can either be imported as `results_proteins.csv` or `results.hdf`.

Intensity types AlphaPept either described the raw intensity or the free quantification (LFQ) intensity. By default AlphaStats uses the LFQ-Intensity for the downstream analysis.

Further, AlphaStats will identify "Reverse" - Proteins.

Find more details about the file format here.

```
import alphastats
alphapept_data = alphastats.AlphaPeptLoader(file="testfiles/alphapept_results_proteins.
↪csv")
```

2.1.3 MaxQuant

MaxQuant generates multiple files as output. For the downstream analysis the `proteinGroups.txt` file, containing the aggregated protein intensities is sufficient.

Intensity types MaxQuant annotates different intensity types: raw intensity, label - free quantification (LFQ) intensity and intensity-based absolute quantification (iBAQ) intensity. The default settings are "LFQ intensity [sample]".

Find more details about the file format here

```
import alphastats
maxquant_data = alphastats.MaxQuantLoader(file="testfiles/maxquant_proteinGroups.txt")
```

2.1.4 DIA-NN

For the analysis of DIA-NN output use `report_final.pg_matrix.tsv`. Versions before 1.7. are not supported.

Find more details about the file format here.

```
import alphastats
diann_data = alphastats.DIANNLoader(file="testfiles/diann_report_final.pg_matrix.tsv")
```

2.1.5 FragPipe

Find more details about the file format here.

```
import alphastats
fragpipe_data = alphastats.FragPipeLoader(file="testfiles/fragpipe_combined_proteins.tsv
↪")
```

2.1.6 Spectronaut

Find more details about the file format here.

As default alphastats will use "PG.ProteinGroups" and "PG.Quantity" for the analysis. For an ananlysis on a peptide level the "F.PeakArea" and the peptide sequences ("PEP.StrippedSequence") can be used.

```
import alphastats
spectronaut_data = alphastats.SpectronautLoader(
    file="testfiles/spectronaut/results.tsv",
    intensity_column = "F.PeakArea",
    index_column = "PEP.StrippedSequence"
    )
```

2.1.7 mzTab

Find more details about the file format here.

```
import alphastats
mztab_data = alphastats.mzTabLoader(
    file="testfiles/mztab/test.mztab"
    )
```

2.2 Preparing metadata

To compare samples across various conditions in the downstream analysis, a metadata file in form of a table (excel, csv, tsv) is required. This file should contain a column with the sample IDs (raw file names) matching the sample names annotated in the output file of your proteomics software. Further, information can be provided like disease and various clinical parameters. Examples of metadata files can be found in the testfiles-folder.

2.3 Creating a DataSet

The whole downstream analysis can be perforemd on the alphastats.DataSet. To create the DataSet you need to provide the loader object as well as the metadata.

```
import alphastats

maxquant_data = alphastats.MaxQuantLoader(
    file="testfiles/maxquant_proteinGroups.txt"
)

dataset = alphastats.DataSet(
    loader = maxquant_data,
    metadata_path="../testfiles/maxquant/metadata.xlsx",
    sample_column="sample"
)
```

CHAPTER

THREE

DATA PREPROCESSING

Preprocessing of the data is substantial for the results of the downstream analysis. Data preprocessing available in AlphaStats covers: the removal of contaminants, normalization, imputation and subsetting and removal of samples.

```
DataSet.preprocess(
        remove_contaminations,
        subset,
        normalization,
        imputation,
        remove_samples
)
```

3.1 Removal of contaminations

- Should I remove contaminations/ `remove_contaminations=True`? Generally speaking - Yes (source)

Various proteomics software annotates contaminants differently or not at all. MaxQuant describes contaminations or ProteinGroups that should be removed in `Only identified by site`, `Reverse`, `Potential contaminant`. Likewise MaxQuant, AlphaPept flags spurious proteins as `Reverse`.

In addition, AlphaStats identifies contaminations based on the contamination library created by Frankenfield et al. 2022.

3.2 Normalization

Depending on the software and the settings, data could already have been normalized before loading into AlphaStats.

AlphaStats has the following Normalization methods implemented:

- **Z-Score Normalization (Standardization)**: Centers the protein intensity of each sample, meaning scaling the variance to 1.
- **Quantile Normalization**: Aims to correct technical bias by adjusting the distribution of protein intensities for each sample. This normalization method is suitable when it is assumed that only a small portion of the protein expression varies among certain conditions, while the majority of the proteome remains stable (Dubois et al., 2022).
- **Linear Normalization**
- **Variance Stabilization Transformation**

Note It has been shown that normalizing the data first and then imputing the data performs better, than the other way around (Karpievitch et al. 2012). This preprocessing order is also acquired in AlphaStats (unless preprocessing is done in several steps).

3.3 Imputation

Especially, missing values are challenging when it comes to analyzing proteomic mass spectrometry data. Missing values can either be missing completely at random (MCAR), due to technical limitations or missing not at random (MNAR) meaning that the abundance is below the detection limit of the platform or completely absent.

To deal with missing values, AlphaStats has the following methods implemented:

- **k-nearest neighbors (kNN)**: Missing values are imputed using the mean protein intensity of k-nearest neighbors
- **Random Forest**: Applies the machine learning algorithm random forest and predicts the values of the target variable using specific known target variables as the outcome and other variables as predictors.
- **median**: Replaces missing values using the median of each protein.
- **mean**: Replaces missing values using the mean of each protein.

Overall, random forest-based imputation for mass spectrometry data has shown a high performance among several studies compared to other imputations methods(Kokla et al. 2019, Jin et al. 2021). However, when applying random forest imputation to your dataset, you have to expect a long run time.

3.4 Subset Data

In case the proteomics data contains more samples than the metadata, the proteomics data can be filtered based on the samples present in the metadata using `DataSet.preprocess(subset=True)`.

3.5 Remove Samples

If you want to remove samples from your `DataSet`, outliers for instance you can give a list of sample names `DataSet.preprocess(remove_samples=["sample1", "sample3"])`.

CHAPTER

FOUR

FUNCTIONS

4.1 Preprocessing

- All preprocessing steps can be done with: `preprocess()`
- The information about the preprocessing steps can be accessed any time using `preprocess_print_info()`

4.2 Figures

To generate interactive plots, AlphaStats uses the graphing library Plotly and all plotting methods will return a plotly object. The plotly graphs returned by AlphaStats can be customized. A description on how do customize your plots can be found here

Plot Intensity

- Plot Intensity for indiviual Protein/ProteinGroup `plot_intensity()`
- Plot Intensity distribution for each sample `plot_sampledistribution()`

Dimensionality reduction plots:

- Principal Component Analysis (PCA): `plot_pca()`
- t-SNE: `plot_tsne()`
- UMAP `plot_umap()`

Plot Distance between samples

- Plot correlation matrix `plot_correlation_matrix()`
- Plot Dendrogram `plot_dendrogram()`
- Plot Clustermap `alphastats.DataSet_Plot.Plot.plot_clustermap()`

Volcano Plot

To estimate the differential expression between two groups, the function plot_volcano() either performs a t-test, an ANOVA or a Wald-test using the package diffxpy .

- Volcano Plot `plot_volcano()`

The results of the statistical analysis for the volcano plot will be saved within the plot and can be accessed:

```
plot = DataSet.plot_volcano(column = "disease", group1 = "healthy", group2 = "cancer")
plot.plotting_data
```

Save Figures

The plots will return a plotly object, thus you can use write_image() from plotly. More details on how to save plotly figures you can find here.

4.3 Statistical Analysis

- Perform Differential Expression Analysis a Wald test or t-test diffxpy. `diff_expression_analysis()`
- ANOVA `anova()`
- ANCOVA `ancova()`
- Tukey - test `tukey_test()`

4.4 GO Analysis

The GO Analysis uses the API from aGOtool.

- Characterize foreground without performing a statistical test: `go_characterize_foreground()`
- Gene Ontology Enrichment Analysis with abundance correction: `go_abundance_correction()`
- Gene Ontology Enrichment Analysis without abundance correction: `go_compare_samples()`
- Gene Ontology Enrichement Analysis using a Background from UniProt Reference Proteomes: `go_genome()`

Visualization of GO Analysis results

All GO-analysis functions will return a DataFrame with the results.

- Plot Scatterplot with -log10(p-value) on x-axis and effect size on y-axis. *df.plot_scatter()*
- Plot p-values as Barplot *df.plot_bar*

4.5 Misc

Get an overview over your dataset

- `overview()`
- `preprocess_print_info()`

CHAPTER

FIVE

GO ANALYSIS

Gene Ontology (GO) Enrichment Analyses are widely used to analyze omics-data sets to identify significant enriched GO terms. AlphaStats utilizes aGOtool for enrichment analysis. aGOtool is specially tailored for Mass Spectrometry (MS) based proteomics data. This tool considers the fact that post-translational modifications (PTMs) are more likely to be detected on highly abundant proteins than on low-abundant proteins. The functional enrichment is performed for GO (molecular function, biological process, cellular component), UniProt keywords, KEGG pathways, PubMed publications, Reactome, Wiki Pathways, Interpro domains, PFAM domains, Brenda Tissues and Diseases. The bias correction by aGOtool aims for increased specificity, fewer significantly enriched but more biologically meaningful and accurate enrichment terms Schölz et al. 2015.

The implementation of aGOtool in AlphaStats will allow you to perform the following analysis:

- **Abundance Correction**: Compares two samples (for example healthy vs. controls). As foreground, all positively, associated proteins of the foreground are used. For the background postively associated proteins and their intensity of the background are used.
- **Characterize Foreground**: Display functional annotations of your Protein(s) of interest without performing a statistical test.
- **Compare Samples**: GO Enrichment ANalysis without abundance correction.
- **Genome**: GO Enrichment Analysis using a Background from UniProt Reference Proteomes.

All functions will return a pandas DataFrame with the results.

5.1 Requirements

A GO Enrichment Analysis using Proteomics data is usually performed on a list of proteins with specific PTMs. Currently, AlphaStats offers the option to load the *evidence.txt* file from *MaxQuant*. This file will be used to extract proteins with PTMs when performing a GO analysis. In case there is no information about PTMs available, a list of upregulated proteins in form of UniProt protein accession numbers can be passed to the functions.

More details about the GO Analysis can be found here:

- aGOtool
- Publication: Schölz et al. 2015

CHAPTER

SIX

API REFERENCE FOR ALPHASTATS

All important features of `alphastats` are documented.

6.1 File Loader

6.1.1 AlphaPeptLoader

class AlphaPeptLoader(*file*, *intensity_column='[sample]_LFQ'*, *index_column='Unnamed: 0'*, *sep=','*, ***kwargs*)

Loader for AlphaPept outputfiles https://github.com/MannLabs/alphapept

6.1.2 DIANNLoader

class DIANNLoader(*file*, *intensity_column='[sample]'*, *index_column='Protein.Group'*, *sep='\t'*, ***kwargs*)

Loader for DIA-NN output files https://github.com/vdemichev/DiaNN

6.1.3 FragPipeLoader

class FragPipeLoader(*file*, *intensity_column='[sample] MaxLFQ Intensity '*, *index_column='Protein'*, *gene_names_column='Gene Names'*, *confidence_column='Protein Probability'*, *sep='\t'*, ***kwargs*)

Loader for FragPipe-Philosopheroutputfiles https://fragpipe.nesvilab.org/docs/tutorial_fragpipe_outputs.html#combined_proteintsv

6.1.4 MaxQuantLoader

class MaxQuantLoader(*file*, *intensity_column='LFQ intensity [sample]'*, *index_column='Protein IDs'*, *gene_names_column='Gene names'*, *filter_columns=['Only identified by site', 'Reverse', 'Potential contaminant']*, *confidence_column='Q-value'*, *evidence_file=None*, *sep='\t'*, ***kwargs*)

Loader for MaxQuant outputfiles

6.1.5 SpectronautLoader

class SpectronautLoader(*file*, *intensity_column='PG.Quantity'*, *index_column='PG.ProteinGroups'*, *sample_column='R.FileName'*, *gene_names_column='PG.Genes'*, *filter_qvalue=True*, *qvalue_cutoff=0.01*, *sep='\t'*)

Loader for Spectronaut outputfiles

6.1.6 mzTabLoader

class mzTabLoader(*file*, *intensity_column: str = 'protein_abundance_[sample]'*, *index_column: str = 'accession'*)

6.2 DataSet

6.2.1 DataSet

class DataSet(*loader*, *metadata_path=None*, *sample_column=None*)

Analysis Object

ancova(*protein_id: str*, *covar: Union[str, list]*, *between: str*) → DataFrame

Analysis of covariance (ANCOVA) with on or more covariate(s). Wrapper around = https://pingouin-stats.org/generated/pingouin.ancova.html

Parameters

- **protein_id** (*str*) – ProteinID/ProteinGroup - dependent variable
- **covar** (*str or list*) – Name(s) of column(s) in metadata with the covariate.
- **between** (*str*) – Name of column in data with the between factor.

Returns

ANCOVA summary:

- `'Source'`: Names of the factor considered
- `'SS'`: Sums of squares
- `'DF'`: Degrees of freedom
- `'F'`: F-values
- `'p-unc'`: Uncorrected p-values
- `'np2'`: Partial eta-squared

Return type

pandas.Dataframe

anova(*column: str*, *protein_ids='all'*, *tukey: bool = True*) → DataFrame

One-way Analysis of Variance (ANOVA)

Parameters

- **column** (*str*) – A metadata column used to calculate ANOVA
- **protein_ids** (*str or list, optional*) – ProteinIDs to calculate ANOVA for - dependend variable either ProteinID as string, several ProteinIDs as list or "all" to calculate ANOVA for all ProteinIDs. Defaults to "all".

- **tukey** (*bool, optional*) – Whether to calculate a Tukey-HSD post-hoc test. Defaults to True.

Returns

- `'Protein ID'`: ProteinID/ProteinGroup
- `'ANOVA_pvalue'`: p-value of ANOVA
- `'A vs. B Tukey test'`: Tukey-HSD corrected p-values (each combination represents a column)

Return type
pandas.DataFrame

batch_correction(*batch: str*)

Correct for technical bias/batch effects Behdenna A, Haziza J, Azencot CA and Nordor A. (2020) pyComBat, a Python tool for batch effects correction in high-throughput molecular data using empirical Bayes methods. bioRxiv doi: 10.1101/2020.03.17.995431

Parameters
batch (*str*) – column name in the metadata describing the different batches

create_matrix()

Creates a matrix of the Outputfile, with columns displaying features (Proteins) and rows the samples.

diff_expression_analysis(*group1: Union[str, list], group2: Union[str, list], column: str = None, method: str = 'ttest', perm: int = 10, fdr: float = 0.05*) → DataFrame

Perform differential expression analysis doing a a t-test or Wald test. A wald test will fit a generalized linear model.

Parameters

- **column** (*str*) – column name in the metadata file with the two groups to compare
- **group1** (*str/list*) – name of group to compare needs to be present in column or list of sample names to compare
- **group2** (*str/list*) – name of group to compare needs to be present in column or list of sample names to compare
- **method** (*str,optional*) – statistical method to calculate differential expression, for Wald-test 'wald', paired t-test 'paired-ttest'. Default 'ttest'

Returns

pandas Dataframe with foldchange, foldchange_log2 and pvalue for each ProteinID/ProteinGroup between group1 and group2.

- `'Protein ID'`: ProteinID/ProteinGroup
- `'pval'`: p-value of the ProteinID/ProteinGroup
- `'qval'`: multiple testing - corrected p-value
- `'log2fc'`: log2(foldchange)
- `'grad'`: the gradient of the log-likelihood
- `'coef_mle'`: the maximum-likelihood estimate of coefficient in liker-space
- `'coef_sd'`: the standard deviation of the coefficient in liker-space
- `'ll'`: the log-likelihood of the estimation

Return type
pandas.DataFrame

go_abundance_correction(*bg_sample*, *fg_sample=None*, *fg_protein_list=None*)

Gene Ontology Enrichement Analysis with abundance correction. Using the API connection from a GO tool: https://agotool.org

For the analysis modified proteins in the foreground sample are compared with proteins and their intensity of the background sample. In case there is no information about PTMs in the dataset a list of enriched proteins in the foreground can be loaded. This list of Protein IDs can be obtaint by performing a differential expression analysis or a ANOVA.

Parameters

- **fg_sample** (*str*) – name of foreground sample
- **bg_sample** (*str*) – name of background sample
- **fg_protein_list** (*list, optional*) – list of enriched protein ids in the foreground sample. Defaults to None.

Returns
DataFrame * `'rank'`: The rank is a combination of uncorrected p value and effect size (based on s value). It serves to highlight the most interesting results and tries to emphasize the importance of the effect size. * `'term'`: A unique identifier for a specific functional category. * `'description'`: A short description (or title) of a functional term. * `'p value corrected'`: p value without multiple testing correction, stemming from either Fisher's exact test or Kolmorov Smirnov test (only for "Gene Ontology Cellular Component TEXTMINING", "Brenda Tissue Ontoloy", and "Disease Ontology" since these are based on a continuous score from text mining rather than a binary classification). * `'effect size'`: Proportion of the Foregrounda nd the Background * `'description'`: A short description (or title) of a functional term. * `'year'`: Year of the scientific publication. * `'over_under'`: Overrepresented (o) or underrepresented (u). * `'s_value'`: The s value is a combination of (minus log) p value and effect size. * `'ratio_in_foreground'`: The ratio in the ForeGround is calculated by dividing the number of positive associations for a given term by the number of input proteins (protein groups) for the Foreground. * `'ratio_in_background'`: The ratio in the BackGround is analogous to the above ratio in the FG, using the associations for the background and Background input proteins instead. * `'foreground_count'`: The ForeGround count consists of the number of all positive associations for the given term (i.e. how many proteins are associated with the given term). * `'foreground_n'`: ForeGround n is comprised of the number of input proteins for the Foreground. * `'background_count'`: The BackGround count is analogous to the "FG count" for the Background. * `'background_n'`: BackGround n is analogous to "FG n". * `'foreground_ids'`: ForeGround IDentifierS are semicolon separated protein identifers of the Forground that are associated with the given term. * `'background_ids'`: BackGround IDentifierS are analogous to "FG IDs" for the Background. * `'etype'`: Short for "Entity type", numeric internal identifer for different functional categories.

Return type
pandas.DataFrame

go_characterize_foreground(*protein_list*, *tax_id=9606*)

Display existing functional annotations for your protein(s) of interest. No statistical test for enrichment is performed. Using the API connection from a GO tool: https://agotool.org

Parameters

- **tax_id** (*int, optional*) – NCBI taxon identifier used as background. Defaults to 9606 (=Homo sapiens).

- **protein_list** (*list*) – list of enriched protein ids in the foreground sample.

Returns

DataFrame * `'rank'`: The rank is a combination of uncorrected p value and effect size (based on s value). It serves to highlight the most interesting results and tries to emphasize the importance of the effect size. * `'term'`: A unique identifier for a specific functional category. * `'description'`: A short description (or title) of a functional term. * `'p value corrected'`: p value without multiple testing correction, stemming from either Fisher's exact test or Kolmorov Smirnov test (only for "Gene Ontology Cellular Component TEXTMINING", "Brenda Tissue Ontoloy", and "Disease Ontology" since these are based on a continuous score from text mining rather than a binary classification). * `'effect size'`: Proportion of the Foregrounda nd the Background * `'description'`: A short description (or title) of a functional term. * `'year'`: Year of the scientific publication. * `'over_under'`: Overrepresented (o) or underrepresented (u). * `'s_value'`: The s value is a combination of (minus log) p value and effect size. * `'ratio_in_foreground'`: The ratio in the ForeGround is calculated by dividing the number of positive associations for a given term by the number of input proteins (protein groups) for the Foreground. * `'ratio_in_background'`: The ratio in the BackGround is analogous to the above ratio in the FG, using the associations for the background and Background input proteins instead. * `'foreground_count'`: The ForeGround count consists of the number of all positive associations for the given term (i.e. how many proteins are associated with the given term). * `'foreground_n'`: ForeGround n is comprised of the number of input proteins for the Foreground. * `'background_count'`: The BackGround count is analogous to the "FG count" for the Background. * `'background_n'`: BackGround n is analogous to "FG n". * `'foreground_ids'`: ForeGround IDentifierS are semicolon separated protein identifers of the Forground that are associated with the given term. * `'background_ids'`: BackGround IDentifierS are analogous to "FG IDs" for the Background. * `'etype'`: Short for "Entity type", numeric internal identifer for different functional categories.

Return type

pandas.DataFrame

go_compare_samples(*fg_sample*, *bg_sample*)

Gene Ontology Enrichement Analysis without abundance correction. Using the API connection from a GO tool: https://agotool.org

Parameters

- **fg_sample** (*str*) – name of the foreground sample
- **bg_sample** (*str*) – name of the background sample

Returns

DataFrame * `'rank'`: The rank is a combination of uncorrected p value and effect size (based on s value). It serves to highlight the most interesting results and tries to emphasize the importance of the effect size. * `'term'`: A unique identifier for a specific functional category. * `'description'`: A short description (or title) of a functional term. * `'p value corrected'`: p value without multiple testing correction, stemming from either Fisher's exact test or Kolmorov Smirnov test (only for "Gene Ontology Cellular Component TEXTMINING", "Brenda Tissue Ontoloy", and "Disease Ontology" since these are based on a continuous score from text mining rather than a binary classification). * `'effect size'`: Proportion of the Foregrounda nd the Background * `'description'`: A short description (or title) of a functional term. * `'year'`: Year of the scientific publication. * `'over_under'`: Overrepresented (o) or underrepresented (u). * `'s_value'`: The s value is a combination of (minus log) p value and effect size. * `'ratio_in_foreground'`: The ratio in the ForeGround is calculated by dividing the number of positive associations for a given term by the number of input proteins (protein groups) for the Foreground. * `'ratio_in_background'`:

The ratio in the BackGround is analogous to the above ratio in the FG, using the associations for the background and Background input proteins instead. * 'foreground_count': The ForeGround count consists of the number of all positive associations for the given term (i.e. how many proteins are associated with the given term). * 'foreground_n': ForeGround n is comprised of the number of input proteins for the Foreground. * 'background_count': The BackGround count is analogous to the "FG count" for the Background. * 'background_n': BackGround n is analogous to "FG n". * 'foreground_ids': ForeGround IDentifierS are semicolon separated protein identifers of the Forground that are associated with the given term. * 'background_ids': BackGround IDentifierS are analogous to "FG IDs" for the Background. * 'etype': Short for "Entity type", numeric internal identifer for different functional categories.

Return type

pandas.DataFrame

`go_genome`(*tax_id=9606*, *fg_sample=None*, *protein_list=None*)

Gene Ontology Enrichement Analysis using a Background from UniProt Reference Proteomes. Using the API connection from a GO tool: https://agotool.org

Parameters

- **tax_id** (*int, optional*) – NCBI taxon identifier used as background. Defaults to 9606 (=Homo sapiens).
- **fg_sample** (*str, optional*) – name of sample used as foreground. Defaults to None.
- **protein_list** (*list, optional*) – list of enriched protein ids in the foreground sample. Defaults to None.

Returns

DataFrame * 'rank': The rank is a combination of uncorrected p value and effect size (based on s value). It serves to highlight the most interesting results and tries to emphasize the importance of the effect size. * 'term': A unique identifier for a specific functional category. * 'description': A short description (or title) of a functional term. * 'p value corrected': p value without multiple testing correction, stemming from either Fisher's exact test or Kolmorov Smirnov test (only for "Gene Ontology Cellular Component TEXTMINING", "Brenda Tissue Ontoloy", and "Disease Ontology" since these are based on a continuous score from text mining rather than a binary classification). * 'effect size': Proportion of the Foregrounda nd the Background * 'description': A short description (or title) of a functional term. * 'year': Year of the scientific publication. * 'over_under': Overrepresented (o) or underrepresented (u). * 's_value': The s value is a combination of (minus log) p value and effect size. * 'ratio_in_foreground': The ratio in the ForeGround is calculated by dividing the number of positive associations for a given term by the number of input proteins (protein groups) for the Foreground. * 'ratio_in_background': The ratio in the BackGround is analogous to the above ratio in the FG, using the associations for the background and Background input proteins instead. * 'foreground_count': The ForeGround count consists of the number of all positive associations for the given term (i.e. how many proteins are associated with the given term). * 'foreground_n': ForeGround n is comprised of the number of input proteins for the Foreground. * 'background_count': The BackGround count is analogous to the "FG count" for the Background. * 'background_n': BackGround n is analogous to "FG n". * 'foreground_ids': ForeGround IDentifierS are semicolon separated protein identifers of the Forground that are associated with the given term. * 'background_ids': BackGround IDentifierS are analogous to "FG IDs" for the Background. * 'etype': Short for "Entity type", numeric internal identifer for different functional categories.

Return type

pandas.DataFrame

load_metadata(*file_path*)

Load metadata either xlsx, txt, csv or txt file

Parameters
file_path (*str*) – path to metadata file

overview()

Print overview of the DataSet

plot_clustermap(***kwargs*)

plot_correlation_matrix(*method='pearson'*)

Plot Correlation Matrix

Parameters

- **method** (*str, optional*) – orrelation coefficient "pearson", "kendall" (Kendall Tau correlation)
- **"spearman"** (*or*) –

Returns
Correlation matrix

Return type
plotly.graph_objects._figure.Figure

plot_dendrogram(***kwargs*)

plot_imputed_values()

plot_intensity(*protein_id, group=None, subgroups=None, method='box', add_significance=False, log_scale=False, compare_preprocessing_modes=False*)

Plot Intensity of individual Protein/ProteinGroup

Parameters

- **ID** (*str*) – ProteinGroup ID
- **group** (*str, optional*) – A metadata column used for grouping. Defaults to None.
- **subgroups** (*list, optional*) – Select variables from the group column. Defaults to None.
- **method** (*str, optional*) – Violinplot = "violin", Boxplot = "box", Scatterplot = "scatter" or "all". Defaults to "box".
- **add_significance** (*bool, optional*) – add p-value bar, only possible when two groups are compared. Defaults False.
- **log_scale** (*bool, optional*) – yaxis in logarithmic scale. Defaults to False.

Returns
Plotly Plot

Return type
plotly.graph_objects._figure.Figure

plot_pca(***kwargs*)

plot_sampledistribution(*method='violin'*, *color=None*, *log_scale=False*)

Plot Intensity Distribution for each sample. Either Violin or Boxplot

Parameters

- **method** (*str, optional*) – Violinplot = "violin", Boxplot = "box". Defaults to "violin".
- **color** (*str, optional*) – A metadata column used to color the boxes. Defaults to None.
- **log_scale** (*bool, optional*) – yaxis in logarithmic scale. Defaults to False.

Returns

Plotly Sample Distribution Plot

Return type

plotly.graph_objects._figure.Figure

plot_samplehistograms()

Plots the Denisty distribution of each sample

Returns

Plotly Graph Object

Return type

plotly

plot_tsne(***kwargs*)

plot_umap(***kwargs*)

plot_volcano(*group1*, *group2*, *column: Optional[str] = None*, *method: str = 'ttest'*, *labels: bool = False*, *min_fc: float = 1.0*, *alpha: float = 0.05*, *draw_line: bool = True*, *perm: int = 100*, *fdr: float = 0.05*, *compare_preprocessing_modes: bool = False*, *color_list: list = []*)

Plot Volcano Plot

Parameters

- **column** (*str*) – column name in the metadata file with the two groups to compare
- **group1** (*str/list*) – name of group to compare needs to be present in column or list of sample names to compare
- **group2** (*str/list*) – name of group to compare needs to be present in column or list of sample names to compare
- **method** (*str*) – "anova", "wald", "ttest", "SAM" Defaul ttest.
- **labels** (*bool*) – Add text labels to significant Proteins, Default False.
- **alpha** (*float,optional*) – p-value cut off.
- **min_fc** (*float*) – Minimum fold change.
- **draw_line** (*boolean*) – whether to draw cut off lines.
- **perm** (*float,optional*) – number of permutations when using SAM as method. Defaults to 100.
- **fdr** (*float,optional*) – FDR cut off when using SAM as method. Defaults to 0.05.
- **color_list** (*list*) – list with ProteinIDs that should be highlighted.
- **compare_preprocessing_modes** (*bool*) – Will iterate through normalization and imputation modes and return a list of VolcanoPlots in different settings, Default False.

Returns
Volcano Plot

Return type
plotly.graph_objects._figure.Figure

preprocess(*log2_transform: bool = True*, *remove_contaminations: bool = False*, *subset: bool = False*, *normalization: str = None*, *imputation: str = None*, *remove_samples: list = None*)

Preprocess Protein data

Removal of contaminations:

Removes all observations, that were identified as contaminations.

Normalization:

“zscore”, “quantile”, “linear”, “vst”

Normalize data using either zscore, quantile or linear (using l2 norm) Normalization.

Z-score normalization equals standaridzation using StandardScaler: https://scikit-learn.org/stable/modules/generated/sklearn.preprocessing.StandardScaler.html

Variance stabilization transformation uses: https://scikit-learn.org/stable/modules/generated/sklearn.preprocessing.PowerTransformer.html

For more information visit. Sklearn: https://scikit-learn.org/stable/modules/generated/sklearn.preprocessing.normalize.html

Imputation:

“mean”, “median”, “knn” or “randomforest” For more information visit:

SimpleImputer: https://scikit-learn.org/stable/modules/generated/sklearn.impute.SimpleImputer.html

k-Nearest Neighbors Imputation: https://scikit-learn.org/stable/modules/impute.html#impute

Random Forest Imputation: https://scikit-learn.org/stable/auto_examples/impute/plot_iterative_imputer_variants_comparison.html https://scikit-learn.org/stable/modules/generated/sklearn.ensemble.RandomForestRegressor.html#sklearn.ensemble.RandomForestRegressor

Parameters

- **remove_contaminations** (*bool, optional*) – remove ProteinGroups that are identified as contamination.
- **log2_transform** (*bool, optional*) – Log2 transform data. Default to True.
- **normalization** (*str, optional*) – method to normalize data: either “zscore”, “quantile”, “linear”. Defaults to None.
- **remove_samples** (*list, optional*) – list with sample ids to remove. Defaults to None.
- **imputation** (*str, optional*) – method to impute data: either “mean”, “median”, “knn” or “randomforest”. Defaults to None.
- **subset** (*bool, optional*) – filter matrix so only samples that are described in metadata found in matrix. Defaults to False.

preprocess_print_info()

Print summary of preprocessing steps

reset_preprocessing()

Reset all preprocessing steps

tukey_test(*protein_id: str*, *group: str*, *df: DataFrame = None*) → DataFrame

Calculate Pairwise Tukey-HSD post-hoc test Wrapper around: https://pingouin-stats.org/generated/pingouin.pairwise_tukey.html#pingouin.pairwise_tukey

Parameters

- **protein_id** (*str*) – ProteinID to calculate Pairwise Tukey-HSD post-hoc test - dependend variable
- **group** (*str*) – A metadata column used calculate pairwise tukey
- **df** (*pandas.DataFrame, optional*) – Defaults to None.

Returns

- `'A'`: Name of first measurement
- `'B'`: Name of second measurement
- `'mean(A)'`: Mean of first measurement
- `'mean(B)'`: Mean of second measurement
- `'diff'`: Mean difference (= mean(A) - mean(B))
- `'se'`: Standard error
- `'T'`: T-values
- `'p-tukey'`: Tukey-HSD corrected p-values
- `'hedges'`: Hedges effect size (or any effect size defined in

`effsize`) * `'comparison'`: combination of measurment * `'Protein ID'`: ProteinID/ProteinGroup

Return type

pandas.DataFrame

CHAPTER

SEVEN

INDICES AND TABLES

- genindex
- modindex
- search

PYTHON MODULE INDEX

a

alphastats.DataSet, 16
alphastats.loader.AlphaPeptLoader, 15
alphastats.loader.DIANNLoader, 15
alphastats.loader.FragPipeLoader, 15
alphastats.loader.MaxQuantLoader, 15
alphastats.loader.mzTabLoader, 16
alphastats.loader.SpectronautLoader, 16

INDEX

A

AlphaPeptLoader (*class in alphastats.loader.AlphaPeptLoader*), 15
alphastats.DataSet
    module, 16
alphastats.loader.AlphaPeptLoader
    module, 15
alphastats.loader.DIANNLoader
    module, 15
alphastats.loader.FragPipeLoader
    module, 15
alphastats.loader.MaxQuantLoader
    module, 15
alphastats.loader.mzTabLoader
    module, 16
alphastats.loader.SpectronautLoader
    module, 16
ancova() (*DataSet method*), 16
anova() (*DataSet method*), 16

B

batch_correction() (*DataSet method*), 17

C

create_matrix() (*DataSet method*), 17

D

DataSet (*class in alphastats.DataSet*), 16
DIANNLoader (*class in alphastats.loader.DIANNLoader*), 15
diff_expression_analysis() (*DataSet method*), 17

F

FragPipeLoader (*class in alphastats.loader.FragPipeLoader*), 15

G

go_abundance_correction() (*DataSet method*), 18
go_characterize_foreground() (*DataSet method*), 18
go_compare_samples() (*DataSet method*), 19
go_genome() (*DataSet method*), 20

L

load_metadata() (*DataSet method*), 21

M

MaxQuantLoader (*class in alphastats.loader.MaxQuantLoader*), 15
module
    alphastats.DataSet, 16
    alphastats.loader.AlphaPeptLoader, 15
    alphastats.loader.DIANNLoader, 15
    alphastats.loader.FragPipeLoader, 15
    alphastats.loader.MaxQuantLoader, 15
    alphastats.loader.mzTabLoader, 16
    alphastats.loader.SpectronautLoader, 16
mzTabLoader (*class in alphastats.loader.mzTabLoader*), 16

O

overview() (*DataSet method*), 21

P

plot_clustermap() (*DataSet method*), 21
plot_correlation_matrix() (*DataSet method*), 21
plot_dendrogram() (*DataSet method*), 21
plot_imputed_values() (*DataSet method*), 21
plot_intensity() (*DataSet method*), 21
plot_pca() (*DataSet method*), 21
plot_sampledistribution() (*DataSet method*), 21
plot_samplehistograms() (*DataSet method*), 22
plot_tsne() (*DataSet method*), 22
plot_umap() (*DataSet method*), 22
plot_volcano() (*DataSet method*), 22
preprocess() (*DataSet method*), 23
preprocess_print_info() (*DataSet method*), 23

R

reset_preprocessing() (*DataSet method*), 23

S

SpectronautLoader (*class in alphastats.loader.SpectronautLoader*), 16

T

tukey_test() (*DataSet method*), 23
