# Supplementary material for: AlphaPeptStats: an open-source Python package for automated and scalable statistical analysis of mass spectrometry-based proteomics
Source: Bioinformatics. 2023 Aug 1;39(8):btad461. doi: 10.1093/bioinformatics/btad461 (PMC10415174; doi:10.1093/bioinformatics/btad461)
Supplement: btad461_Supplementary_Data [file btad461_supplementary_data.zip › supplementary_notebook_1_liu_2019.html]

liu\_2019


# Plasma proteome profiling discovers novel proteins associated with non-alcoholic fatty liver disease¶

Liu et al. 2019

In this notebook we want to reproduce the results from Liu et al. The aim is to identify proteins associated with non-alcoholic fatty liver disease (NAFLD).

In [1]:

```
import alphastats
import plotly.io as pio
pio.renderers.default = "plotly_mimetype+notebook"
```

In [2]:

```
!pip show alphastats
```

```
Name: alphastats
Version: 0.6.2
Summary: An open-source Python package for automated and scalable statistical analysis of mass spectrometry-based proteomics
Home-page: https://github.com/MannLabs/alphastats
Author: Mann Labs
Author-email: elena.krismer@gmail.com
License: Apache
Location: /Users/drq441/Documents/GitHub/alphastats
Requires: anndata, click, combat, data_cache, diffxpy, kaleido, numba, numba-stats, numpy, openpyxl, pandas, pingouin, plotly, pyteomics, scikit-learn, scipy, sklearn_pandas, statsmodels, streamlit, swifter, tables, tqdm, umap-learn
Required-by:
```

We are going to load the `proteinGroups.txt` and the corresponding metadatafile. You can find the data here or on ProteomeXchange PXD011839.
To load the proteomis data you need to create a loader object using `alphastats.MaxQuantLoader`. The whole downstream analysis will be performed on a `alphastats.DataSet`. To create the DataSet you need to provide the loader object as well as the metadata.

In [3]:

```
# we are going to load the proteomics data
loader = alphastats.MaxQuantLoader(
    file = "../testfiles/maxquant/proteinGroups.txt", 
    intensity_column="LFQ intensity [sample]",
    # for indexing we are going to use the gene name columm
    # it is adivsed to use the ProteinIDs for indexing as these are unique
    index_column="Gene names",
    gene_names_column=None
)
dataset = alphastats.DataSet(
    loader = loader, 
    metadata_path="../testfiles/maxquant/metadata.xlsx", 
    sample_column="sample"
)
```

```
DataSet has been created.
Attributes of the DataSet can be accessed using: 
DataSet.rawinput:	 Raw Protein data.
DataSet.mat:	Processed data matrix with ProteinIDs/ProteinGroups as columns and samples as rows. All computations are performed on this matrix.
DataSet.metadata:	Metadata for the samples in the matrix. Metadata will be matched with DataSet.mat when needed (for instance Volcano Plot).
```

The metadata contains pretty long names, in the first step we are going to replace the names with abbreviations to make the analysis more straightforward.

In [4]:

```
new_names = {
    "non-alcoholic fatty liver disease": "NAFLD",
    "type 2 diabetes mellitus": "T2DM",
    "type 2 diabetes mellitus|non-alcoholic fatty liver disease": "T2DM+NAFLD"
}
dataset.metadata["disease"].replace(new_names, inplace=True)
```

For preprocessing we are going to use quantile normalization, k-Nearest Neighbour imputation and remove contanminants.

In [5]:

```
dataset.preprocess(
    log2_transform=False,
    remove_contaminations=True, 
    subset=True, 
    imputation="knn", 
    normalization="quantile"
)
```

In [6]:

```
dataset.preprocessing_info
```

Out[6]:

```
{'Raw data number of Protein Groups': 2596,
 'Matrix: Number of ProteinIDs/ProteinGroups': 1811,
 'Matrix: Number of samples': 48,
 'Intensity used for analysis': 'LFQ intensity [sample]',
 'Log2-transformed': False,
 'Normalization': 'quantile',
 'Imputation': 'k-Nearest Neighbor',
 'Contaminations have been removed': True,
 'Contamination columns': ['Only identified by site',
  'Reverse',
  'Potential contaminant',
  'contamination_library'],
 'Number of removed ProteinGroups due to contaminaton': 98}
```

## Plot Volcano¶

Which proteins are significantly higher or lower in intensity comparing Diabetes mellitus Type 2 and Diabetes mellitus Type 2 + non-alcoholic fatty liver disease?

In [7]:

```
volcano_plot = dataset.plot_volcano(
    column="disease", # column in metadata
    group1="T2DM",
    group2="T2DM+NAFLD",
    labels=True, # add label to significantly enriched proteins
    alpha=0.001 # cutoff for pvalue
)
volcano_plot.show(renderer = "png")
```

```
Calculating Students t-test...
```

## Plot Intensity¶

Boxplot of intensity values of T2DM and T2DM+NAFLD of idenitified enriched/downregulated proteins.

In [8]:

```
for protein in ["LGALS3BP","AFM","SERPINC1","CTSD;HEL-S-130P","ALDOB"]:
    plot = dataset.plot_intensity(
        method="all",
        protein_id=protein, 
        group="disease", # column in metadata
        subgroups=["T2DM","T2DM+NAFLD"], # we only want to see T2DM and T2DM+NAFLD
        add_significance=True # add bar with pvalue
        )
    plot.show(renderer = "png")
```

Jupyter Notebook can be found:
https://github.com/MannLabs/alphapeptstats/blob/main/nbs/liu\_2019.ipynb
