# Supplementary material for: AlphaPeptStats: an open-source Python package for automated and scalable statistical analysis of mass spectrometry-based proteomics
Source: Bioinformatics. 2023 Aug 1;39(8):btad461. doi: 10.1093/bioinformatics/btad461 (PMC10415174; doi:10.1093/bioinformatics/btad461)
Supplement: btad461_Supplementary_Data [file btad461_supplementary_data.zip › supplementary_notebook_2_ramus_2016.html]

ROC


In [1]:

```
import alphastats
import pandas as pd
import numpy as np
```

In [2]:

```
!pip show alphastats
```

```
Name: alphastats
Version: 0.6.2
Summary: An open-source Python package for automated and scalable statistical analysis of mass spectrometry-based proteomics
Home-page: https://github.com/MannLabs/alphastats
Author: Mann Labs
Author-email: elena.krismer@gmail.com
License: Apache
Location: /Users/drq441/Documents/GitHub/alphastats
Requires: anndata, click, combat, data_cache, diffxpy, kaleido, numba, numba-stats, numpy, openpyxl, pandas, pingouin, plotly, pyteomics, scikit-learn, scipy, sklearn_pandas, statsmodels, streamlit, swifter, tables, tqdm, umap-learn
Required-by:
```

# Example: Standardized spiked proteomics Data PXD001819¶

Data from Ramus et al. (2016), contains samples spiked with various amounts of recombinant proteins. We are going to compare different preprocessing settings using the MaxQuant (LFQ) data. More details about the dataset can be found here.

In [3]:

```
# Get list of UPS-spiked proteins
original_df = pd.read_excel("Supplementary Table 1.xlsx", sheet_name="LFQ MaxQuant (7)")
original_df =original_df[original_df['Majority protein IDs'].notna()]
ups_proteins = original_df[(original_df['Species'] == "UPS")]['Majority protein IDs'].to_list()
```

In [4]:

```
# load data
loader = alphastats.MaxQuantLoader(
    file= "ProteinGroups.txt",
    index_column="Majority protein IDs",
    intensity_column = "[sample]",
    filter_columns=[]
)
                                
ds = alphastats.DataSet(
    loader = loader,
    metadata_path = "metadata.csv",
    sample_column = "sample"  
)
```

```
DataSet has been created.
Attributes of the DataSet can be accessed using: 
DataSet.rawinput:	 Raw Protein data.
DataSet.mat:		Processed data matrix with ProteinIDs/ProteinGroups as columns and samples as rows. All computations are performed on this matrix.
DataSet.metadata:	Metadata for the samples in the matrix. Metadata will be matched with DataSet.mat when needed (for instance Volcano Plot).
```

## Volcano Plot¶

In [5]:

```
column="condition_replicate"
group1="50Vs5_A"
group2="50Vs5_B"
method="sam"
perm=10
color_list=ups_proteins

draw_line=False,
labels:bool=False
min_fc:float=1.0
alpha:float=0.05
draw_line:bool=False
fdr:float=0.05
```

In [6]:

```
volcano_plot = ds.plot_volcano(
    group1=group1,
    group2=group2,
    column=column,
    method=method,
    labels=labels,
    min_fc=min_fc,
    alpha=alpha,
    draw_line=draw_line,
    perm=perm, 
    fdr=fdr,
    color_list=color_list
)
volcano_plot
```

```
Calculating t-test and permuation based FDR (SAM)...
```

*Fig. Volcano plot (− log10(p-value) of the significance analysis of microarray (sam) test versus protein log2(fold change))is shown. The graph illustrate the quantitative results for the UPS1 proteins, colored in pink quantified in the binary comparison 5 fmol/μg versus 50 fmol/μg. Gray dots correspond to yeast proteins quantified in all samples.*

## Compare different preprocessing modes¶

## ROC Curve¶

Proteins meeting the defined criteria were categorized as variants. Among these, proteins belonging to the UPS1 group were considered true positives (TP), while those originating from yeast were regarded as false positives (FP). On the other hand, proteins classified as non-variants based on the criteria were considered true negatives (TN) if they were from yeast, and false negatives (FN) if they were UPS1.

To assess the sensitivity of various imputation and normalization algorithms we used the condition 5 fmol/μg versus 50 fmol/μg.

In [7]:

```
results = ds.plot_volcano(
    group1=group1,
    group2=group2,
    column=column,
    method=method,
    labels=labels,
    min_fc=min_fc,
    alpha=alpha,
    draw_line=draw_line,
    perm=perm, 
    fdr=fdr,
    color_list=color_list, 
    compare_preprocessing_modes=True
)
```

```
All preprocessing steps are reset.
Normalization vst, Imputation mean
Data has been log2-transformed.
Calculating t-test and permuation based FDR (SAM)... 
	
All preprocessing steps are reset.
Normalization vst, Imputation median
Data has been log2-transformed.
Calculating t-test and permuation based FDR (SAM)... 
	
All preprocessing steps are reset.
Normalization vst, Imputation knn
Data has been log2-transformed.
Calculating t-test and permuation based FDR (SAM)... 
	
All preprocessing steps are reset.
Normalization vst, Imputation randomforest
Data has been log2-transformed.
Calculating t-test and permuation based FDR (SAM)... 
	
All preprocessing steps are reset.
Normalization zscore, Imputation mean
Data has been log2-transformed.
Calculating t-test and permuation based FDR (SAM)... 
	
All preprocessing steps are reset.
Normalization zscore, Imputation median
Data has been log2-transformed.
Calculating t-test and permuation based FDR (SAM)... 
	
All preprocessing steps are reset.
Normalization zscore, Imputation knn
Data has been log2-transformed.
Calculating t-test and permuation based FDR (SAM)... 
	
All preprocessing steps are reset.
Normalization zscore, Imputation randomforest
Data has been log2-transformed.
Calculating t-test and permuation based FDR (SAM)... 
	
All preprocessing steps are reset.
Normalization quantile, Imputation mean
Data has been log2-transformed.
Calculating t-test and permuation based FDR (SAM)... 
	
All preprocessing steps are reset.
Normalization quantile, Imputation median
Data has been log2-transformed.
Calculating t-test and permuation based FDR (SAM)... 
	
All preprocessing steps are reset.
Normalization quantile, Imputation knn
Data has been log2-transformed.
Calculating t-test and permuation based FDR (SAM)... 
	
All preprocessing steps are reset.
Normalization quantile, Imputation randomforest
Data has been log2-transformed.
Calculating t-test and permuation based FDR (SAM)... 
	
```

In [8]:

```
from sklearn.metrics._ranking import roc_curve
from sklearn.metrics._ranking import auc
import matplotlib.pyplot as plt
import seaborn as sns
sns.set_palette('Set1', n_colors=12)

def get_plot_from_predictions(y_true, y_pred,name, color=None):
    fpr, tpr, _ = roc_curve(y_true, y_pred,)
    roc_auc = auc(fpr, tpr)
    plot = plt.plot(
        fpr, 
        tpr, 
        label="{}, AUC={:.3f}".format(name, roc_auc)
    )
    return plot   

for result in results:
    res = result.plot.plotting_data
    res['TP'] = res['Majority protein IDs'].str.contains('UPS')
    res['FP'] = ~res['Majority protein IDs'].str.contains('UPS')
    res.replace([np.inf, -np.inf], np.nan, inplace=True)
    resna = res[~res['pval'].isna()]
    resna = res[~res['tval'].isna()]
    
    norm_meth = result.plot.preprocessing["Normalization"]
    imp_meth = result.plot.preprocessing["Imputation"]
    preprocessing_mode = f"Normalization: {norm_meth}, Imputation: {imp_meth}"
          
    probs = resna['pval']
    probs = -probs
        
    plot = get_plot_from_predictions(
        y_true = resna['TP'],
        y_pred = probs,
        name = preprocessing_mode
    )
        
plt.plot([0, 1], [0, 1], "k--", label="chance level (AUC = 0.5)")
plt.axis("square")
plt.xlabel("False Positive Rate")
plt.ylabel("True Positive Rate")
plt.title('pval')
plt.legend(bbox_to_anchor=(1.04, 1), loc="upper left")
plt.show()
```

*Fig. ROC curves plotted from MaxQuant LFQ intensity dataset (PXD001819) using the comparison 5 fmol/μg versus 50 fmol/μg. Overlaid ROC curves for different preprocessing settings. Proteins were classified as variant by filtering on the p-value threshold. Abbreviation: vst: Variance Stabilization Transformation*
