# Supplementary material for: AlphaPeptStats: an open-source Python package for automated and scalable statistical analysis of mass spectrometry-based proteomics
Source: Bioinformatics. 2023 Aug 1;39(8):btad461. doi: 10.1093/bioinformatics/btad461 (PMC10415174; doi:10.1093/bioinformatics/btad461)
Supplement: btad461_Supplementary_Data [file btad461_supplementary_data.zip › supplement_material.pdf]

Supplementary Material

**Data Import and Data types**	
Label-free quantitative proteomics data	✓
TMT, SILAC	✗
protein-centric	✓
peptide-centric	✗
Phosphoproteomics	✗
Immunoproteomics	✗
Metaproteomics	✗
**Quality control**	
Chromatography profile	✗
Sample distribution	✓
**Data preprocessing**	
Normalization	✓
Imputation	✓
Filtering	✓
Batch correction	✓
**Data Analysis**	
Differential Expression Analysis	✓
Hierarchical Clustering	✓
Dimensionality Reduction	✓
Gene Ontology term analysis	✓
Time series analysis	✗
Protein Network Reconstruction	✗
**Supported Operating Systems**	
macOS	✓
Windows	✓
Linux	✓

Supplementary Table 1: Overview of supported functionalities by AlphaPeptStats indicated by ✔ (implemented) and ✘ (not implemented) .

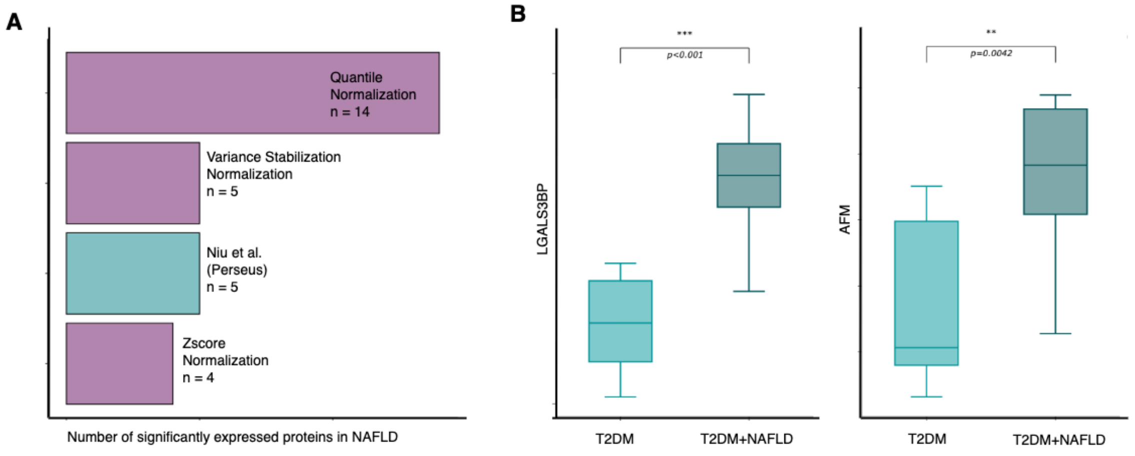

Supplementary Figure 1: A. Systematic optimization of several normalization methods on a NAFLD dataset leading to different numbers of significantly expressed proteins with up to 14 when using Quantile Normalization. B. Example plots of LGALS3BP and AFM showing significant expression. Abbreviations: T2DM: Diabetes-Mellitus Type 2, NAFLD: non-alcoholic fatty liver disease

To exemplify the capabilities of AlphaPeptStats, we applied it to our recently published study on non-alcoholic liver disease (NAFLD) (**Niu *et al*., 2019**). Using default settings, this successfully reproduced the same significantly differentially expressed proteins as previously reported, except for one (PIGR). As PIGR was validated as a biomarker in that study, we investigated the reason for its disappearance. We found that this biomarker was just below our stringent cutoff criteria due to too many missing values. Next, we leveraged the automatic parameter optimization and systematically investigated how the different preprocessing steps would maximize the number of differentially expressed proteins. This uncovered 9 additional biomarkers for NAFLD compared to the original five when applying quantile normalization, shown in **Supplement Figure 1A**. Exploration of the additional biomarkers indicated a plausible connection to NAFLD (Suppl. Table 2). Exemplary plots generated with AlphaPeptStats of the enriched proteins LGALS3BP and AFM in NAFLD are presented in **Supplement Figure 1B**.

Protein	Full name	Biological Process	Supporting Literature
C9	Complement component C9	Immune system regulation and inflammation	(Subudhi et al., 2022)
SERPINC1	Serpin peptidase inhibitor, clade C (antithrombin), member 1	Coagulation cascade	(Bell et al., 2010)
ANPEP	Alanyl aminopeptidase	Angiogenesis	(Martinou et al., 2022)
ITIH4	Inter-alpha-trypsin inhibitor heavy chain 4	Immune system regulation and inflammation	(Martinou et al., 2022)
APOB	Apolipoprotein B	Cholesterol and triglyceride balance	(Bell et al., 2010)
C3	Complement component C3	Immune system regulation and inflammation	(Ogresta et al., 2022)
FGA	Fibrinogen A alpha	Coagulation cascade	(Ogresta et al., 2022)
FGG	Fibrinogen gamma chain	Coagulation cascade	(Ogresta et al., 2022)
AMBP	Alpha-1-Microglobulin/Bikunon	Host-virus interaction	(Berezin et al., 2023)
CFH	Complement factor H	Immune system regulation and inflammation	(Ogresta et al., 2022)
SERPINF2	Serpin Family F member 2	Coagulation cascade	(Bell et al., 2010)
AMF	Autocrine motility factor	Gluconeogenesis, Glycolisis	(Lin et al., 2020)
1TP5BP1	Tumor Protein P53 Binding Protein 1	DNA damage, repair	(Akazawa et al., 2019)
GPLD1	Glycosylposphatidylinositol sepcific phospholipase D1	Lipid metabolism	(Yuan et al., 2008)
LGALS3BP	lectin	Cell adhesion	(Wood et al., 2017)

Supplementary Table 2. Significantly altered proteins in non-alcoholic fatty liver disease (NAFLD) , identified with AlphaPeptStats.

Supplementary References

Akazawa, Y., Nakashima, R., Matsuda, K., Okamaoto, K., Hirano, R., Kawasaki, H., Miuma, S., Miyaaki, H., Malhi, H., Abiru, S., Itoh, M., Kondo, H., Fukuoka, J., Nakao, K., & Nakashima, M. (2019). Detection of DNA damage response in nonalcoholic fatty liver disease via p53-binding protein 1 nuclear expression. Modern Pathology, 32(7), 997–1007. https://doi.org/10.1038/s41379-019-0218-8

Bell, L. N., Theodorakis, J. L., Vuppalanchi, R., Saxena, R., Bemis, K. G., Wang, M., & Chalasani, N. (2010). Serum proteomics and biomarker discovery across the spectrum of nonalcoholic fatty liver disease. Hepatology, 51(1), 111–120. https://doi.org/10.1002/hep.23271

Berezin, A. A., Obradovic, Z., Berezina, T. A., Boxhammer, E., Lichtenauer, M., & Berezin, A. E. (2023). Cardiac Hepatopathy: New Perspectives on Old Problems through a Prism of Endogenous Metabolic Regulations by Hepatokines. Antioxidants, 12(2), 516. https://doi.org/10.3390/antiox12020516

Lin, H., Zhu, L., Baker, S. S., Baker, R. D., & Lee, T. (2020). Secreted phosphoglucose isomerase is a novel biomarker of nonalcoholic fatty liver in mice and humans. Biochemical and Biophysical Research Communications, 529(4), 1101–1105. https://doi.org/10.1016/j.bbrc.2020.06.126

Martinou, E., Pericleous, M., Stefanova, I., Kaur, V., & Angelidi, A. M. (2022). Diagnostic Modalities of Non-Alcoholic Fatty Liver Disease: From Biochemical Biomarkers to Multi-Omics Non-Invasive Approaches. Diagnostics, 12(2), 407. https://doi.org/10.3390/diagnostics12020407

Nakamura, N., Hatano, E., Iguchi, K., Sato, M., Kawaguchi, H., Ohtsu, I., Sakurai, T., Aizawa, N., Iijima, H., Nishiguchi, S., Tomono, T., Okuda, Y., Wada, S., Seo, S., Taura, K., Uemoto, S., & Ikegawa, M. (2019). Elevated levels of circulating ITIH4 are associated with hepatocellular carcinoma with nonalcoholic fatty liver disease: From pig model to human study. BMC Cancer, 19(1), 621. https://doi.org/10.1186/s12885-019-5825-8

Ogresta, D., Mrzljak, A., Cigrovski Berkovic, M., Bilic-Curcic, I., Stojsavljevic-Shapeski, S., & Virovic-Jukic, L. (2022). Coagulation and Endothelial Dysfunction Associated with NAFLD: Current Status and Therapeutic Implications. Journal of Clinical and Translational Hepatology, 10(2), 339–355. https://doi.org/10.14218/JCTH.2021.00268

Subudhi, S., Drescher, H. K., Dichtel, L. E., Bartsch, L. M., Chung, R. T., Hutter, M. M., Gee, D. W., Meireles, O. R., Witkowski, E. R., Gelrud, L., Masia, R., Osganian, S. A., Gustafson, J. L., Rwema, S., Bredella, M. A., Bhatia, S. N., Warren, A., Miller, K. K., Lauer, G. M., & Corey, K. E. (2022). Distinct Hepatic Gene-Expression Patterns of NAFLD in Patients With Obesity. Hepatology Communications, 6(1), 77–89. https://doi.org/10.1002/hep4.1789

Wood, G. C., Chu, X., Argyropoulos, G., Benotti, P., Rolston, D., Mirshahi, T., Petrick, A., Gabrielson, J., Carey, D. J., DiStefano, J. K., Still, C. D., & Gerhard, G. S. (2017). A multi-component classifier for nonalcoholic fatty liver disease (NAFLD) based on genomic, proteomic, and phenomic data domains. Scientific Reports, 7(1), 43238. https://doi.org/10.1038/srep43238

Yuan, X., Waterworth, D., Perry, J. R. B., Lim, N., Song, K., Chambers, J. C., Zhang, W., Vollenweider, P., Stirnadel, H., Johnson, T., Bergmann, S., Beckmann, N. D., Li, Y., Ferrucci, L., Melzer, D., Hernandez, D., Singleton, A., Scott, J., Elliott, P., … Mooser, V. (2008). Population-Based Genome-wide Association Studies Reveal Six Loci Influencing Plasma Levels of Liver Enzymes. The American Journal of Human Genetics, 83(4), 520–528. https://doi.org/10.1016/j.ajhg.2008.09.012

Niu, L. et al. (2019) Plasma proteome profiling discovers novel proteins associated with non-alcoholic fatty liver disease. Mol Syst Biol, 15
